# Application of Smart Packaging on the Preservation of Different Types of Perishable Fruits

**DOI:** 10.3390/foods14111878

**Published:** 2025-05-26

**Authors:** Andreas Panou, Dimitrios G. Lazaridis, Ioannis K. Karabagias

**Affiliations:** Department of Food Science & Technology, School of Agricultural Sciences, University of Patras, 30100 Agrinio, Greece; andreapanou@gmail.com (A.P.); up1076474@ac.upatras.gr (D.G.L.)

**Keywords:** perishable fruits, intelligent packaging, active packaging, preservation, shelf-life extension, challenges

## Abstract

The packaging of perishable products, such as fruits, contributes to their preservation during storage and safe transportation. The use of suitable packaging materials contributes to forming a desirable atmosphere inside the package so that the level of respiration, transpiration, and ethylene emission can be kept low. However, it would be useful for consumers to know relevant information on the deterioration rate of different types of fruit (tree fruits, berries, stone fruits, and aggregate accessory fruits). The technology of intelligent and active packaging systems (smart packaging) enables the provision of information related to the deterioration rate of fruits to consumers and, in parallel, extends the shelf life of fruits and other plant-based foods, maintaining a high quality. Intelligent packaging systems include biosensors and gas sensors, along with microbial, freshness, and time–temperature indicators. On the other hand, the active packaging system includes the use of moisture, odor, and gas absorbers, along with antioxidant and antimicrobial agents to maintain the quality of plant-based foods and extend their shelf life. This review article aims to make an in-depth evaluation of the most relevant literature on this topic by highlighting the challenges, trends, and future directions related to different types of fruits.

## 1. Introduction

Smart packaging is a food packaging application that can be applied in both synthetic polymers and biodegradable ones. It has the advantage of food chemical monitoring and preserves food freshness for a long period, considering also that when including biodegradable films and coatings the environmental impact is low compared to conventional plastic packaging. Smart packaging is mainly divided into two categories: (i) intelligent packaging and (ii) active packaging. The intelligent packaging system includes the usage of biosensors, gas sensors and microbial freshness, and time–temperature indicators. The active packaging system includes the use of moisture, odor, and gas absorbers, and antioxidant and antimicrobial agents [1]. The intelligent and active packaging systems protect food from the effects of various physical, chemical, and biological agents. Active packaging introduces various active components (i.e., chitosan, cellulose, essential oils, organic acids, fungicides, bacteriocins, antibiotics, enzymes, alcohols, whey protein, lipids, etc.) inside the packaging material. These active components have both antioxidant and antimicrobial activity and can become active either in synthetic (plastic) polymers or in edible films and coatings [2]. Edible films and coatings are well known for protecting perishable fruits (i.e., avocado, banana, strawberries, etc.) [3] from deterioration by retarding dehydration, suppressing respiration, improving texture, retaining flavor profile, and reducing the growth of microorganisms [2]. The use of edible films and coatings as carriers of additives to extend the shelf life of perishable foods has been widely studied in recent years. Similarly, edible films and coatings, along with nanocomposites carrying antimicrobials, are a promising tool for decreasing the risk of pathogenic bacteria [3].

The common characteristics of intelligent and active packaging systems are the extension of shelf-life of packaged food, the reduction of food waste, the increase of consumer acceptability, the maintenance of quality, and safety insurance. The main goal of an active packaging system is the improvement of packaging functionality by the addition of supplementary elements into the packaging material. This process maintains the food’s freshness and prolongs its shelf life. The active packaging system may thus delay food quality changes and warn of possible problems. The smart packaging system provides improved results related to food quality. The parameters used in smart packaging system quality indices are the pH and temperature changes, volatile compounds, and several other indices of freshness [4].

According to Holman et al. [5], the communication of intelligent packaging can reduce the energy consumption that happens through the cold chain, the number of preservatives, and unnecessary food waste.

Smart packaging offers the advantage of reducing food waste, and it can also protect, control, and maintain the quality and prolong the shelf-life of packaged food [6]. In the United States, the Food and Drug Administration (FDA) regulates active and intelligent packaging. The goal of the addition of chemicals or scavengers to food packaging is the reduction of the spoilage rate and the maintenance of the sensory characteristics of the food. According to the Code of Federal Regulations (C.F.R.), some food additives are added to packaging for the improvement of physical or technical properties of the final product [7]. This regulation states that the level of these additives should not exceed the amount that can cause physical or technical changes in the food contact material, for which no limitations have been established. In active and intelligent packaging systems, if materials do not migrate to the food or have a technical effect on the food, there are no problems concerning the regulation of their amounts. However, any additional migrants, decomposition byproducts, or impurities must also be considered.

In the European Union, active and intelligent packaging was initially regulated by Regulation (EC) No 450/2009 [European Commission]; however, this directive is no longer in force. It has been replaced by Commission Regulation (EU) No 10/2011 of 14 January 2011 on plastic materials and articles intended to come into contact with food, which has a current consolidated version from March 2025 [8].

According to these regulations, the specific substances responsible for active and/or intelligent activity must be included on the European Community list of eligible substances, with some limited exceptions. The active and/or intelligent regulations establish that “active materials and articles are intended to increase the shelf-life or to maintain or improve the condition of packaged food” and “intelligent materials and articles monitor the condition of packaged food or the environment surrounding the food”. According to the updated (EC) No 10/2011, active and intelligent materials can be promoted to the market if they meet the following criteria:

(i). They must be suitable for the intended purpose.
(ii). They must comply with general safety requirements for all food-contact materials and with specific requirements for all active and intelligent materials and articles.
(iii). They must comply with the composition, labeling, and declaration requirements of the regulation.

The migration level of substances that are not in direct contact with food or their surrounding environment is equal to 0.01 mg/kg for each group of substances that have been classified proportional to the structural and toxicological characteristics. The European Food Safety Authority (EFSA) is responsible for the declaration of the safety of active and intelligent substances before their use in the EU.

The safety assessments that must be examined are mainly associated with dietary exposure to chemicals because of:

(a) The migration of the active and/or intelligent substance(s).
(b) The migration of their degradation and/or reaction products.
(c) Their toxicological properties.

The safety aspects of active and intelligent packaging are regulated by Regulation 1935/2004. The Framework Regulation (EC) 1935/2004 contains all materials and articles intended to come into contact with food. There is also a specific directive (Directive 2002/72/EC) that includes plastic materials. Any active and intelligent packaging systems that do not include plastic materials are not subject to Directive 2002/72/EC. These materials must comply with the national legislation of some countries, e.g., Germany and the Netherlands. Until now, there is no specific directive or regulation for the active/intelligent component in active and intelligent packaging, and they must comply with the Framework Regulation (EC) 1935/2004. The packaging of the active or intelligent components and the active and intelligent components are subjected to Article 3 and Article 4, respectively. Article 3 of the Framework Regulation (EC) 1935/2004 defines that the amounts of constituents transferred to food should not exceed the limit which could lead to the following:

(i). Harms to human health.
(ii). Undesirable changes in chemical composition and sensory characteristics.

Also, consumers should not be misled by labelling, advertising, or the presentation of material or articles.

The main requirements of Article 4 of the Framework Regulation, which referred to active and intelligent materials, are the following:

(i). Active materials may cause changes in sensory quality or composition of foods, provided that the changes comply with the community or national provisions applicable to food.
(ii). The released substances from active packaging must be authorized, and their use must comply with the relevant community provisions applicable to food.
(iii). The organoleptic changes caused by active materials should not mask the spoilage of food and mislead consumers.
(iv). The information about intelligent materials should not refer to the condition of the food, which could mislead consumers.
(v). The labelling must enable the discrimination of non-edible parts.
(vi). The labelling must indicate that the materials are active and/or intelligent.

Considering the above, and mostly the updated regulation [8], this review article aimed to make an in-depth evaluation of the most relevant literature on the use of smart packaging in the shelf-life estimation and quality control of selected types of fruit (i.e., tree fruits, berries, stone fruits, etc.) by highlighting the challenges, trends, and future directions related to this topic. To the best of our knowledge, limited review works have been carried out over the last 5 years on this subject, which comprises the novelty of the current article.

## 2. Application of a Smart Packaging System to Fruits

The deterioration rate of fruits (and vegetables) depends on several factors, such as the respiration and transpiration rate, ethylene production/action rate, the injuries of fruits, and the growth of several yeasts and molds. For these reasons, fruits and/or vegetables need special preservation conditions inside the packaging, such as temperature, gas composition, and humidity [9]. The main goals after the harvest of fruits (and vegetables) are the inhibition of respiration, transpiration, ripening, and senescence rate. The film packaging of a packaged fruit or vegetable must permit gas exchange [10]. For the reduction of respiration, transpiration, and ethylene production rate, the reduction in temperature is crucial. It must be noted that tropical and subtropical fruits (tomato, pineapple, banana, mango, avocado, olive, papaya, cherimoya, star fruit, etc.) are subjected to physiological damage when exposed to low temperature (5–15 °C) [11]. Devices that monitor several temperature-dependent negative changes in fresh and minimally processed fruits and vegetables are time–temperature indicators (TTI), which show the accumulated time-temperature data of a product during storage [12].

Ethylene is a gaseous unsaturated hydrocarbon produced by the metabolism of fruits and vegetables and is responsible for the increase of ripening rate, but it also increases the senescence rate and causes quality degradation of fruits and vegetables. For the elimination of ethylene production, ethylene absorbers such as potassium permanganate, alumina, and silica in the form of sachets are placed inside the packaging [13,14]. Ethylene absorbers reduce weight loss, inhibit the ripening rate, and preserve the firmness. Apart from ethylene, oxygen also increases the oxidative changes of fruits and vegetables, and it enables the growth of yeasts and molds [15,16]. For the reduction of all undesirable oxidative changes of oxygen, as well as the protection of β-carotene oxidation, oxygen absorbers are used [17,18]. Cruz and his coworkers [19] stated that the rate of loss of ascorbic acid and the oxidation rate in packaged oranges, along with oxygen scavengers, was significantly lower. Other factors that act as indicators of deterioration are the production of undesirable volatile compounds (aldehydes, amines, and sulfides) and the determination of ripeness state [20]. The ripeness state of strawberries can be determined by the application of methyl red-based packaging. The production of esters causes a reduction in pH, which causes a color change [21].

### 2.1. Tree Fruits

#### Apple

Apples (*Malus domestica*) belong to the Rosaceae family, and their fleshy parenchyma tissue consists of cuticle, epidermal, and the hypodermal layer. Apple trees are the most widely cultivated species of the genus *Malus* and originated in Central Asia. The cultivation of apples was done for thousands of years in Eurasia and was spread to North America by European colonists. Apple fruit consists of 77.8–88.5% water, 7.5–16.4% sugars, 0.18–0.72% crude proteins, 0.1–0.42% minerals, 1–47 mg/100 g ascorbic acid and several organic flavor compounds, markers of maturation and ripening such as aldehydes (acetaldehyde, 2-methylbutanal, 3-methylbutanal, hexanal, trans-2-hexenal), esters (ethyl acetate, ethyl propionate, ethyl 2-methylpropionate, ethyl butyrate, ethyl 2-methyl butyrate, 3-methylbutyl acetate, 2-methylbutyl acetate, 1-propyl butyrate, ethyl pentanoate, amyl acetate, ethyl hexanoate), alcohols (ethanol, 1-butanol, 2-methyl-1-butanol, hexanol), and ethylene [22,23,24,25,26,27]. These compounds act as fruit freshness, ripeness, and quality of maturity indicators, measured using fluorescence sensors [28], electronic noses [29,30,31], and colorimetric chemosensors [32]. Naked-eye-detected chemosensors are very important on-packaging indicators because they provide information on the stage of maturity and ripeness. In apple ripening, colorimetric sensors using ethylene emission as a marker were applied [32,33,34]. However, they exhibited some disadvantages because of their high cost and low stability against humidity and UV light. Aldehyde-sensitive colorimetric sensors using pH indicators have also been applied. A sensor of glutaraldehyde and formaldehyde detection is also very useful [35,36]. The packaging of apples into plastic films with polyvinyl alcohol PVA containing ethylene scavengers extended the storability of packaged apples [37].

For the determination of the ripeness stage of pear and several other fruits and vegetables, a ripeness indicator with sensors capable of detecting aromas and volatiles named RipeSense has been developed [4]. The color change of the sensor (red–orange–yellow) depends on the state of ripeness. For the determination of apple ripeness stage, a color-based ripeness indicator has been developed [32]. This ripeness indicator consists of molybdenum (VI), which partially decreases to molybdenum (V) when it reacts with ethylene. These reactions change color from white/yellow to blue. Another label-based colorimetric sensor detects aldehyde through the nucleophilic addition reaction between aldehyde and hydroxide using methyl red as an indicator [38]. The label changes color from yellow to orange, and finally to red when the indicator is exposed to ripening apples.

The action of ethylene of a fresh product inside the package can be neutralized by the application of ethylene scavenging systems. This is performed through the adsorption or chemical alteration of ethylene hormone by active compounds such as potassium permanganate, sodium permanganate, and titanium dioxide that cause oxidation of ethylene to carbon dioxide and water. Also, activated carbon, charcoal, zeolites, clays, and metal–organic frameworks can be used for the ethylene absorption. The use of ethylene scavengers in packaging acts positively in the shelf life of apples [39,40,41]. 1-Methylcyclopropene (MCP) acts as an ethylene inhibitor in active packaging. MCP is non-toxic, has high efficiency, competes with ethylene for ethylene receptors (AdERS1a, Ad-ETR2, and Ad-ETR3), and also prevents the expression of several transcription factors (Ad-ERF4, Ad-ERF6, Ad-ERF10, and Ad-ERF14). The competition between MCP and ethylene receptors contributes to the inhibition of over-ripening in post-harvest stages. Inhibition of ethylene synthesis can also be achieved by 1-pentylcyclopropene (1-PentCP) and 1-octylcyclopropene (1-OCP), which are structural analogs of 1-MCP. 1-MCP also plays other physiological roles that do not have such clear mechanisms [13].

The application of pure and encapsulated potassium metabisulphite in the ratio of 1:1 plays an important role in the preservation of apples, inhibition of enzymatic browning, and the growth of harmful microorganisms. The packaging of Gala apples in polyvinyl chloride (PVC) films with the active compound (potassium metabisulfite) reduces the microbial load and the extension of enzymatic browning and increases their shelf life. A high microbiological stability over 20 days was reported after the packaging of apples in PVC film with 2% potassium metabisulfite [42]. The incorporation of antimicrobial agents in the form of essential oils of palmarosa and star anise, included in cyclodextrin complex, into a double-bottom tray suppressed the growth of *Penicillium expansum* inoculated into apples and increased their shelf life. A reduction in weight loss by 50% and a reduction in loss of firmness by 25% were also attained [43].

### 2.2. Berries

#### 2.2.1. Kiwifruit

Kiwifruit or Chinese gooseberry is an edible berry of the genus *Actinidia* [44,45] and is cultivated mostly in central and eastern China. Kiwifruit (*Actinidia deliciosa*) are oval, and they have a thin, fuzzy, fibrous, tart but edible light brown skin and light green or golden flesh with edible seeds [46]. Kiwifruit have a soft texture and a sweet and characteristic flavor. Kiwi is a fruit that has a unique flavor and high nutritional value. The volatile compounds of Kiwi are isobutyl butyrate, ethyl 2-furoate, ethyl valerate, propyl butyrate, ethyl hexanoate, 4-terpene alcohols, benzyl alcohol, oxime-methoxy-phenyl, and 4-isopropyl toluene [47]. Most of the above-mentioned volatile compounds are related to deterioration of fruit freshness and ripening, and they are identified efficiently by electronic nose [29,30,48], fluorescence sensors [28], and colorimetric chemical sensors [32]. The use of these sensors is limited because of their high costs and complexity of operating requirements. Today, the freshness of fruits and vegetables is monitored mainly by colorimetric sensors, which comprise indicators in real-time analysis and provide information on the quality of the product through color changes [49]. Shao et al. [50] studied the correlation between the aldehyde emission of Kiwi and the color changes of a colorimetric film consisting of ethyl cellulose/polyvinyl alcohol (EC/PVA), incorporated with poly (ethylene glycol) bis(3-aminopropyl) terminated (amine-PEG) and methyl red. They observed that, during Kiwi ripening, the color of the film turned from yellow to orange and finally to red. The color changes of the film were correlated with the changes in Kiwi freshness parameters [50].

Kiwifruit packaged along with scavenger sachets containing charcoal and palladium chloride at 20 °C for two days presented higher firmness and better maintenance of quality in comparison with the control sample [51]. The increase of shelf life of Kiwifruit can be accomplished by the use of ethylene absorbers [41]. Potassium permanganate has been used as an ethylene gas scavenger in the monitoring of ripeness [52]. The application of lipase TTI (time–temperature indicator) was suitable for the monitoring of kiwifruit ripeness [53]. Also, Oh et al. [53] reported that the Arrhenius activation energy (Ea) of kiwifruit firmness was correlated with the Ea of ethylene gas production. The incorporation of copper (I) complex into the packaging film and the use of a bathophenanthroline-based palladium (Pd) complex have also been used for ethylene detection during the ripening of packaged Kiwi [34,54].

#### 2.2.2. Avocado

The avocado (*Persea americana*), commonly known as alligator pear or avocado pear, is an evergreen tree, and it is included in the laurel family (*Lauraceae*). Avocado trees grow in Mexico and Costa Rica. Avocado is cultivated in the tropical and Mediterranean climates of many countries [55]. Avocado is a large berry that contains a single large seed and consists of oil, vitamins, and chemical compounds with high antioxidant properties [56]. A ripe avocado has smooth, buttery, golden-green flesh, green, brown, purplish, or black skin, and its shape may be similar to that of a pear or egg or may be globally proportional to the cultivar. Avocado is harvested unripe and is ripened during storage. Avocado has low storability at room temperature because of its high respiration and ethylene emission rate [57,58]. The shelf life of avocado depends on the amount of ethylene, which can be determined by ethylene absorbers with high sensitivity [41]. Nano zeolite-ammonium molybdate acts as an indicator of avocado ripening. Zeolite is a material that can be used as a matrix for ethylene absorption, along with ammonium molybdate as an ethylene-sensitive color [59]. The color change of the nano zeolite molybdate depends on the quantity of ethylene gas during avocado ripening. So, any information on the progress of ripening can be useful to consumers. The ripeness indicator is placed inside the packaging as a label or printed on packaging materials to monitor the product quality [60]. The function of an indicator label is based on the reaction between the emitted chemical compounds of the product with the materials of the indicator label. This reaction leads to color changes.

Furthermore, a ripeness sensor that can be used in the ripeness monitoring of avocado is the Ripesense [61]. This ripeness monitor was constructed in a New Zealand-based company and is a great solution for ripeness monitoring. The sensor has the characteristic of reacting with volatile compounds of avocado and other fruits. The color of the sensor changes from red (crisp) to orange (firm) and finally to yellow (juicy) during ripening. Iskandar et al. [62] used ammonium molybdate as a color indicator on the ripeness of avocado. They noticed that the changes in the label’s color were related to the degradation of the avocado’s quality. The consumers can easily gain an opinion about the ripening stage by watching the color change of the sensor. The sensor package is constructed of recyclable polyethylene terephthalate (PET) clamshell, which is a hygienic and environmentally friendly packaging solution. In addition, the shelf life of avocado can be extended by the application of ethylene absorbers for 20 days upon post-harvest [41,63].

#### 2.2.3. Banana

Banana is a berry [64] that is produced by several kinds of flowering plants of the genus *Musa*. Banana is elongated, curved, and rich in starch; it has soft flesh covered with a peel, and its color changes when ripe. Also, there are differences in the characteristics of size, color, and firmness. The edible seedless (parthenocarp) cultivated bananas are grown by the plants *Musa acuminata* and *Musa balbisiana*, or their hybrids. The raw banana consists of 75% water, 23% carbohydrates, 1% protein, vitamin B6, vitamin C, manganese, potassium, and dietary fiber.

Unripe bananas stored in 175-gauge and 250-gauge non-perforated HDPE bags with ethylene absorbers exhibited decay on the 15th day of storage, compared to the control sample, which exhibited decay on the 9th day of storage. Also, these bananas presented significantly minimum weight, loss, spoilage, TSS, and pulp/peel ratio throughout storage at 26–29 °C and relative humidity (R.H.) of 58% [65]. The storage of packaged mature ‘Kolikuttu’ bananas with potassium permanganate in low-density polyethylene (LDPE) bags of 75 μm thickness at room temperature (25 ± 2 °C) and R.H. of 85 ± 1% reduced the concentration of ethylene and carbon dioxide and increased the concentration of oxygen. Minimum changes were recorded in firmness, TSS content, weight loss, titratable acidity, and pH. The shelf-life of the analyzed banana samples exceeded 20 days [66]. The application of potassium permanganate and gibberellic acid inhibited the ethylene action and increased the storage life of the banana by up to 18 days [67]. In another study, the presence of Pd/zeolite caused a significant reduction in ethylene concentration during 18 days of storage at 20 ± 2 °C [68]. The application of sachets containing KMnO_4_ on an inert substrate caused a reduction in ethylene production and delayed the senescence of banana. A reduction in weight loss of 2% was also attained compared to the control samples. Moreover, bananas stored with sachets at 14 °C presented less disease severity than the bananas stored with sachets at 20 °C after 16 days of storage. No chilling injury was recorded during storage of bananas at 14 °C [69]. The addition of 5% (*w/w*) TiO_2_ in nanofiber films contributed to the photocatalytic degradation of ethylene and a delay in color change, softening, and postharvest ripening of banana [70].

1-methyl cyclopropane (1-MCP) is a non-toxic and environmentally friendly gaseous four-carbon cyclic olefin, which delays both the biosynthesis and action of ethylene at the nL/L level [71,72,73]. The mechanism of 1-MCP action refers to the binding of 1-MCP to the ethylene receptor site in fruit tissues, thereby inhibiting the effects of ethylene [74,75,76,77,78]. 1-MCP can act effectively even at very low concentrations because of its ten-fold higher affinity for ethylene binding receptors [77]. Also, the expression of genes related to ethylene signaling pathways is significantly inhibited [79]. In bananas, 1-MCP also induced a reduction in the function of enzymes that take part in ethylene synthesis [80]. The application of 1-MCP at a preclimacteric stage of banana reduced the ethylene production rate, the loss of green color, and the production of volatile compounds. Application of 1-MCP 24 h after the propylene treatment and after the initiation of autocatalytic ethylene did not affect the production of ethylene but decreased the color change and the production of volatile compounds [81].

In other studies, the treatment of 1-MCP delayed the ripening of mature-green banana fruits by up to 12 days at 20 °C. This delay was lower when banana fruits were treated before ethylene [82]. Also, the combination of 1-MCP treatment (750 ppb for 24 h) and storage at low temperature (14 °C) decreased the rate of color changes and respiration rate and increased the firmness and shelf-life of banana fruit [81,83,84,85]. In banana cv. ‘Brazil’, the combination of 50 µL/L ethephon with 400 nL/L 1-MCP (16 h) significantly decreased the ripening process of banana fruits without any negative effect on the normal progress of ripening [86]. Many other studies have proved the effect of 1-MCP on the delay of color changes, ripening, respiration process, firmness maintenance, and the prolongation of shelf-life of banana fruit [81,83,84,87,88,89,90,91,92,93,94,95]. The effectiveness of 1-methylcyclopropene (1-MCP) is related to the species, cultivar, active concentration, time of treatment, temperature, and applied method, along with the size and developmental stage and plant maturity of the crop [81,84,96,97,98,99,100,101,102,103].

#### 2.2.4. Tomato

A tomato is an edible berry of the plant. The species of this plant are grown in western South America, Mexico, and Central America. The chemical composition of tomatoes includes proteins, essential amino acids, monounsaturated fatty acids, vitamins, minerals, fiber, carotenoids, and phytosterols [104,105,106,107]. Tomato is a perishable fruit due to its high moisture content [108]. The losses of fresh tomatoes during postharvest are related to handling, storage, and packaging. The most effective way of controlling postharvest losses is smart packaging innovations. The application of active packaging in tomatoes contributes to the increase of their shelf life and safety and the maintenance of their organoleptic characteristics [9,109,110]. The incorporation of chitosan and cinnamic acid into coating forms improved the sensory characteristics, such as firmness and total soluble solids, decreased the weight loss, and extended the shelf life of packaged tomatoes [111]. An extension of the shelf life of tomato for a month was exhibited by the use of an active biodegradable corrugated cardboard tray [109]. The treatment with 0.1% (*v/v*) ethanol increased the content of ascorbic acid, sucrose, and fructose, inhibited ripening, and improved the organoleptic characteristics of cherry tomato during storage [112].

For the reduction of ethylene concentration, ethylene scavengers such as potassium permanganate (KMnO_4_), activated carbon, clay, and zeolites are used. Ethylene scavengers turn the ethylene to acetate and ethanol. Although potassium permanganate has high efficacy in the reduction of ethylene concentration, it has low efficiency as a postharvest tool, and it also harms safety, health, and the environment [113]. According to Mansourbahmani et al. [114], KMnO_4_-promoted nano zeolite has high effectiveness in the reduction of ethylene concentration by approximately 60%. Bailen et al. [115] studied the effect of granular-activated carbon (GAC) alone or impregnated with palladium as a catalyst (GAC-Pd) on some quality characteristics and the shelf life of packaged tomatoes under modified atmosphere packaging [115]. The results showed that tomatoes containing GAC-Pd produced lower amounts of ethylene inside the package compared to the control samples. Also, treated tomatoes exhibited lower weight loss, color evolution and softening, better sweetness, firmness, juiciness, color, odor, and flavor, and lower decay rate compared to control samples. The application of mixed TiO_2_/SiO_2_ (80/20) showed the highest degradation rate of ethylene in mature green tomatoes [116].

Tomato also exhibits high susceptibility in microbial growth. One antimicrobial agent that can act against the growth of microorganisms is the mixture of itaconic acid and chitosan enriched with tomato bioactive extract [117]. Other antimicrobial agents that have been used in the preservation of packaged tomato are silver zeolite, organic acids, spice/herb extract, vitamins C and E, sorbates, chlorine dioxide/sulfur dioxide, and benzoates and propionates [118].

The addition of 20g of NaCl absorbers packaged into LDPE film red-ripe tomatoes at 20 °C prolonged their shelf life by 10–12 days compared to the control sample [119]. Humidity absorbers decrease the humidity level in the packages as a result of the increase in shelf-life of fresh fruits and vegetables [120]. Some of the available humidity absorbers are sorbitol, sodium chloride, potassium chloride (fast humidity absorbers), and bentonite (slower humidity absorbers) [120]. Other active elements that are used for the removal of moisture are silica gel, polyacrylate salts, zeolites, and microporous clays [121]. For the prevention of moisture condensation, a sodium polyacrylate-cotton mixture is used in the form of sachets as a moisture adsorbent [121]. In high relative humidity, the condensation of water vapor occurred on the internal surface of the film packaging. This problem can be solved by the addition of antifogging agents [122]. Factors that should be considered before choosing a suitable moisture absorber are the species, water vapor permeability of packaging materials, storage condition of products, absorption capacity of absorbers, and initial humidity level [123]. Except for humidity absorbers, oxygen absorbers play an important role in the extension of the shelf life of tomatoes. The use of oxygen absorbers increases the shelf life of packaged tomatoes [17].

### 2.3. Stone Fruits

#### 2.3.1. Mango

Mango is a tropical stone fruit produced by the tree *Mangifera indica*. There are two types of modern mango cultivars: the “Indian type” from South Asia and the “Southeast Asian type” from Southeast Asia [124,125]. In the region of Malaysia, there are also other species of the genus *Mangifera* that produce fruits named “mangoes” [126]. Mango presents differences in size, shape, sweetness, skin color, and flesh color (pale yellow, gold, green, or orange) corresponding to its cultivar [55]. The raw mango contains 84% water, 15% carbohydrates, and 1% protein. Mango peel and pulp contain monoterpenes 3-carene, limonene, β-ocimene, myrcene, and α-terpinolene, triterpene lupeol, carotenoids (beta-carotene, lutein, and alpha-carotene), and polyphenols, such as quercetin, kaempferol, gallic acid, caffeic acid, catechins, and tannins [127,128,129,130,131,132,133,134].

Packaged Mango in low-density polyethylene (LDPE) material along with ethylene absorbers presented a nine-times higher shelf life in comparison with packaged Mango in LDPE material without ethylene absorbers [135]. Also, the addition of 6% potassium permanganate in packaged mangoes into polyethylene bags exhibited the lowest weight loss and decay percentage and the highest total soluble solids, vitamin C, and total and reducing sugars, and extended the shelf-life of the product [136]. The application of activated carbon along with potassium permanganate decreased the weight loss, softness, and total soluble solids in mangoes placed in boxes lined with corrugated cardboard [137]. It is believed that potassium permanganate is more effective in liquid form. However, the application of potassium permanganate in liquid form causes problems [138]. Zeolite, alumina beads, vermiculite, or activated carbon are some inert materials that can be used for the absorption of potassium permanganate from a liquid state [57,138,139,140,141].

The application of the electric nose is also very important in the quantification of volatile compounds and the ripening stage of mango [142,143,144,145,146,147,148]. Factors affecting the aroma compounds of mango are environmental conditions, cultivation methods, stage of ripeness, and the handling and storage conditions after its harvest [149]. Volatile compounds that give the characteristic aroma to mango are ethylene and aromatic hydrocarbons (terpene hydrocarbons) [150]. This makes feasible the determination of the optimal harvest date by comparison of the data analysis of volatile compounds with the odor pattern (‘smellprint’) of mango [151]. For electrical nose analysis, the determination of physicochemical and microbiological parameters, such as pH, total soluble solids, weight loss, surface color, firmness, yellowing rate, polyphenol oxidase activity, and total viable counts, is useful.

#### 2.3.2. Sweet Cherry

Sweet cherry (*Prunus avium*) is a non-climacteric sweet, edible stone fruit that is consumed fresh. It contains vitamin A, dietary fiber, and antioxidants, along with small amounts of minerals such as calcium and phosphorus. There are three varieties of sweet cherry: Bing, Rainier, and Queen Anne. The color of these varieties ranges from yellow through red to nearly black. These varieties are characterized by low acidity and a sweet, mildly tart flavor. For the growth of cherries, climates with moderate winter and summer temperatures are required. The most important sweet cherry cultivars in the western United States cover 80% of production and include Bing (the leading cultivar in North America), Van, and Lambert. The other cultivars are used as pollenizers or for the satisfaction of consumers’ demands for large, light-colored, and crisp-fleshed fruits [152].

1-MCP is a chemical cyclic unsaturated hydrocarbon that is used in the gaseous state for the inhibition of ethylene biosynthesis and action, mainly in the preclimacteric stage of climacteric fruits. This results in the delay of ripening and senescence and the prolongation of the shelf life of fruits during cold storage. It must be noted that 1-MCP at high concentrations induced the degradation of non-climacteric fruit quality [153]. The application of 1-MCP at a concentration of 1 μL/L for 24 h in early-season sweet cherries at 1 °C maintained their firmness until the seventh day of storage, while decreasing the severity of physiological disorders at the end of storage life (30 days). In addition, 1-MCP inhibited the gathering of cyanidin-3-O-glucoside for 7 days compared to untreated sweet cherries [152]. Sharma et al. [154] also noticed a better firmness in post-harvest cherries treated with 1-MCP and hexanal sweet cherries [154]. In other studies, no significant changes were reported on the color and firmness of sweet cherries treated with 1-MCP [155,156]. The treatment with 1-MCP greatly delayed the expression of genes related to chlorophyll catabolism in cherry pedicel [157].

### 2.4. Aggregate Accessory Fruits

#### Strawberry

The garden strawberry (*Fragaria × ananassa*) is a widely grown hybrid species of the genus *Fragaria* of the family *Rosaceae*. Strawberry consists of 91% water, 8% carbohydrates, 1% protein, vitamin C, and manganese. Strawberry also contains phytochemical compounds such as agrimoniin–dimeric ellagitannin, ellagic acid, and ellagic acid glycosides; flavonoids, such as anthocyanins, flavanols, and flavonols; and phenolic acids, such as hydroxybenzoic acid and hydroxycinnamic acid [153,157,158,159,160,161]. The ripening monitoring of strawberries uses a simple and cost-efficient ripeness indicator that was designed based on methyl red. Change of color from yellow to red-purple happened after the absorption of methyl red onto bacterial cellulose membrane and through its interactions with volatile acids [21]. The mix of dye-based indicators contains more colors, and they have higher sensitivity to pH change. The use of a freshness indicator gives reliable information on the freshness condition of the product.

1-Methylcyclopropene (1-MCP) has found application as an ethylene action inhibitor in the preclimacteric stage of climacteric fruits. 1-MCP delays the ripening and senescence of fruits and increases their shelf life during cold storage. Although 1-MCP is applied mainly to climacteric fruits, 1-MCP has also been applied to non-climacteric fruits. The treatment of strawberries with 1-MCP and their subsequent storage in air containing 0.1 μL/L ethylene increased their postharvest shelf life in comparison to control samples [162]. The ethylene receptors may have differences in non-climacteric and climacteric fruits [163], and any function related to regulation may differ among ethylene receptors [164]. The inhibitory effect of 1-MCP on strawberry rot has been supported [165]. Strawberries’ firmness and color have also been maintained by treatment with 1-MCP [165]. In both studies, 1-MCP at concentrations of 500 to 1000 ppb increased the disease development rate. 1-MCP negatively affects some of the metabolic pathways of polyphenols, which in turn results in the decrease of the fruit’s defense [162,163]. In our opinion, the higher concentration of 1-MCP results in the oxidation of specific polyphenols by generating oxidized enzymes such as polyphenol oxidase (PPO). However, this is probably related to fruit species/cultivar and storage conditions. It is a challenge for future research.

Table 1 shows the application of active and intelligent packaging systems in the types of fruits discussed above in some representative studies in the recent literature.

## 3. Principles of the Operation of Smart Packaging, Advantages, and Toxicity

As referred to in Table 1, a plethora of active and intelligent packaging systems have been used to prevent and detect changes in the quality characteristics of fruits and vegetables. Of course, every packaging technology has advantages and disadvantages depending on the fruit type (or vegetables) and the operating or processing conditions. More specifically, 1-MCP is one of the most used inhibitors in the food industry for ethylene detection and inhibition, as it affects the post-harvesting quality of fruits and vegetables, while it helps to properly understand the role of ethylene in senescence and ripening processes. 1-MCP has been focused commercially on apple fruit, and it was an important example to illustrate the opportunities and limitations, while it has also been used later in other fruits, such as banana, avocado, and tomato [166]. Some of the advantages attributed to 1-MCP-treated apples are ripening inhibition [167], delay of softening and changes in color [168], inhibited respiration rates [166], and delay in titratable acidity concentrations [169], along with better and more preferred features in sensory analysis by consumers [170]. However, the commercial use of 1-MCP poses numerous challenges for storage operators and growers, because apple is a fruit with numerous cultivars that exhibit different features. As a result, this led to confusion about the treatment variables depending on the cultivar, storage time, and the characteristics expected by the consumers [166,171]. Later, the application of 1-MCP in other fruits and vegetables had similar advantages and disadvantages to apples [166].

Similarly, potassium permanganate (KMnO_4_) is an ethylene scavenging agent with powerful properties. Over the last 50 years, potassium permanganate has been used in active food packaging for fresh products, mainly for climacteric fruits, for ethylene inhibition, ripening delay, chlorophyll degradation, sugar and acidity changes, and weight changes [113]. The application of potassium permanganate in active packaging materials is commonly achieved by the fabrication of it onto microporous mineral particles, which are later placed into sachets to avoid direct contact with food and together in modified atmosphere packaging [113]. Potassium permanganate has been used widely in the preservation of apple [172], banana [173], kiwifruit [174], tomato [114], strawberry [175], and many other fruits [136], all possessing reduced ethylene production, better color and firmness, and slow total acidity decrease. Overall, the benefits of KMnO_4_ in active food packaging have been widely researched [176]. However, even though KMnO_4_ is placed in safe devices to avoid contamination with food and poisoning attributed to KMnO_4_ ingestion, there is a low consumer acceptance, as they fear consuming fresh products packaged with KMnO_4_ particles [113,177]. -based products should be handled carefully due to their harmfulness if swallowed [178], although KMnO_4_ has been used widely in low concentrations as antifungal and antiseptic drugs production [179]. In conclusion, despite the strong ethylene scavenging activity of KMnO_4_ and its performance to preserve the physiological and chemical characteristics of freshly stored products, there is still work to be done regarding consumers’ awareness, to improve their acceptance [113].

Moreover, during recent years, there has been an exponential growth in the development of active food packaging fabricated with metal nanoparticles, such as titanium oxide (TiO_2_), an economic metal oxide with low toxicity and stability, along with antibacterial activity and film mechanical property improvement [180]. In the European Union, TiO_2_ is allowed to be used as a food additive (E 171), while in China it has been widely used as a coloring agent in concentrations up to 10 g/kg [181]. Some reasons why TiO_2_ has been used in active food packaging are (i) the water loss reducing properties, (ii) good gas exchange of the food, (iii) the improved film barrier properties, and (iv) ethylene degradation due to TiO_2_ photocatalytic activity [180].

**Figure 1:**
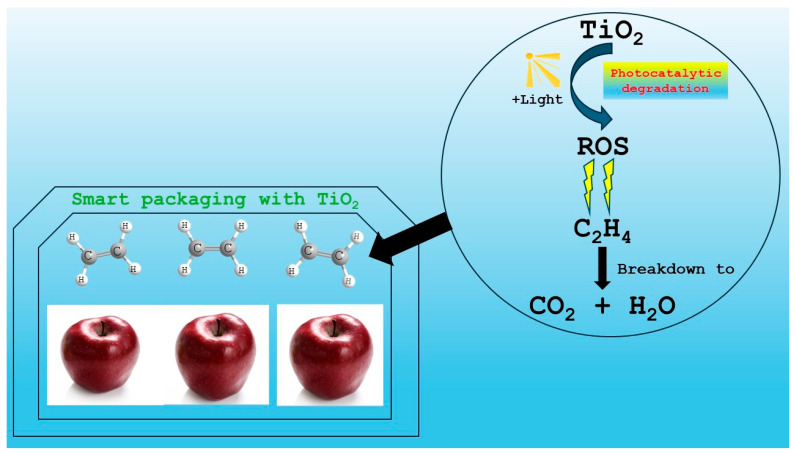
The effect and mechanism action of TiO_2_ in smart packaging.

The difference between TiO_2_ and KMnO_4_ concerning ethylene inhibition is that TiO_2_ decomposes ethylene, utilizing its photocatalytic properties through the electron transfer from TiO_2_ under ultraviolet light, producing ROS and converting ethylene to H_2_O and CO_2_, while KMnO_4_ ethylene inhibition is due to its strong oxidative activity, resulting in the decomposition of ethylene into CO_2_ and H_2_O [13,182]. Concerning the toxicity of TiO_2_ in nanoparticles, in vivo studies have shown that oral consumption can cause heart, spleen, liver, and kidney injury, but its toxicity was weak [181], while other studies reported that the toxicity of TiO_2_ nanoparticles was higher than the traditional bulk particles [183,184], due to their small size and large surface area [185]. Figure 1 describes the influence and mechanism of action of TiO_2_ in smart packaging.

**Figure 2:**
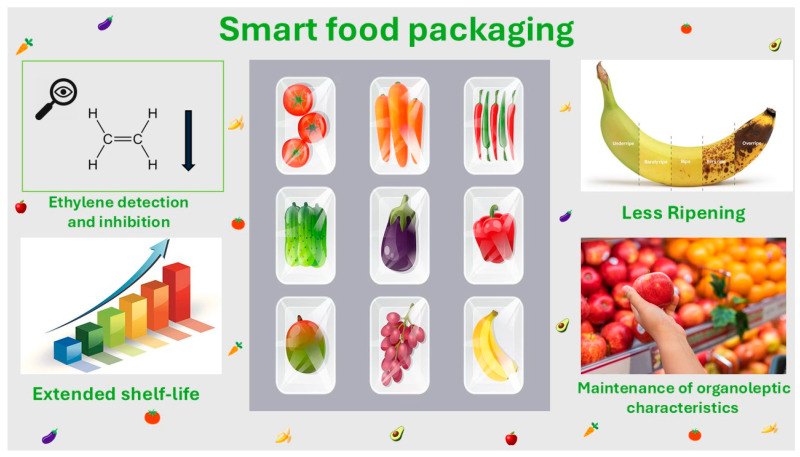
The application and benefits of smart packaging on the preservation of fruits.

Finally, the benefits of active and intelligent packaging utilization are important. Different advantages and disadvantages depend on the packaging material and mechanism of action, but overall, ethylene inhibition and all the results discussed previously by using smart packaging materials on fresh fruits and vegetables are superb. As a result, success in the maintenance of their physiological and chemical characteristics, along with shelf-life extension, can lead to further research studies, proving the importance, effectiveness, and risk for human toxicity of these packaging materials. Figure 2 depicts the benefits of smart packaging applications on the preservation of fruits.

On the other hand, there are limited reports on the toxicity of smart packaging tools, such as ethylene inhibitors, implemented in smart packaging (Table 2). It is surely a field for more exhaustive and consecutive research.

## 4. Conclusions and Future Perspectives

Smart packaging, including active and intelligent packaging, comprises novel technology for the preservation and shelf-life extension of perishable commodities, such as fruits. The topic is gaining more and more attention throughout the global zone, given that food security and food quality are parameters that are strongly correlated with the packaging materials used for food storage and distribution. However, more research is required, especially on the use of ethylene inhibitors or any other ‘‘smart’’ agent implemented in smart packaging, given the scarce data in the literature. We should also not forget the relevant regulations that must be followed and updated. The present review article addresses these aspects, and it is one of the limited available studies in the literature that focuses on this field.

## Figures and Tables

**Table 1:** Application of intelligent and active packaging to several perishable fruits.

Fruits	Intelligent Packaging System	Active Packaging System	References
Apple	Colorimetric sensors using ethylene emission, Aldehyde-sensitive colorimetric sensors using pH indicators, Ripe sense ripeness indicator, Color-based ripeness indicator, Label-based colorimetric sensor using methyl red	Ethylene scavengers, Ethylene inhibitors (1-MCP, 1-PCP, 1-OCP)	[4,13,32,33,34,37,38,39,40,41]
Avocado		Ethylene absorbers with high sensitivity, Nano zeolite-ammonium molybdate, Ripesense, Ammonium Molybdate	[41,59,61,62]
Banana	TiO_2_ nanoparticles	Ethylene absorbers, Ethylene inhibitors (1-MCP)	[41,65,66,70,81,82,83,84,85,87,88,89,90,91,92,93,94,95]
Sweet cherry		Ethylene inhibitors (1-MCP)	[152,154,155,156,157]
Kiwifruit	Electronic nose, Fluorescence sensor, Colorimetric chemical sensor, Lipase TTI (Time-temperature indicator),	Potassium permanganate, Copper (I) complex and bathophenanthroline- based palladium (Pd) complex	[28,29,30,32,34,48,52,53,54]
Mango	Electric nose	Ethylene absorbers	[135,136,137,142,143,144,145,146,147,148]
Strawberry	Ripeness indicator based on methyl red	Ethylene inhibitors (1-MCP)	[21,162,165]
Tomato	Sodium polyacrylate-cotton mixture, Mixed nanoparticles TiO_2_/SiO_2_	Humidity absorbers (sorbitol, sodium chloride, potassium chloride, bentonite, silica gel, polyacrylate salts, zeolites, and microporous clays), Ethylene scavengers such as potassium permanganate (KMnO_4_), activated carbon, clay and zeolites, KMnO_4_-promoted nano zeolite, Granular-activated carbon (GAC), Oxygen absorbers,	[17,113,114,115,116,120,121]

**Table 2:** Toxicity level of ethylene inhibitors used in smart packaging.

Ethylene Inhibitors	Toxicity	References
1-MCP	No	[186]
KMnO_4_	Low	[113]
TiO_2_	Medium	[180]

## Data Availability

The manuscript includes all the relevant data.
